# Afferent-hair cell connectivity as a possible source of spike train irregularity in turtle vestibular bouton afferents

**DOI:** 10.1186/1471-2202-15-S1-P69

**Published:** 2014-07-21

**Authors:** William R Holmes, Janice A Huwe, Michael H Rowe, Ellengene H Peterson

**Affiliations:** 1 Department of Biological Sciences, Neuroscience Program, Ohio University, Athens, OH 45701, USA

## 

Vestibular afferents have been categorized as “regular” or “irregular” depending on the variability of their spike trains [1,2]. It is thought that the difference in regularity among afferents results from differences in the types of voltage-dependent conductances present in the cell. For example afferents may differ in their complements of potassium A, D, and M currents or in currents that produce a small vs. prominent afterhyperpolarization (AHP) [3-5]. Because bouton afferents from turtle utricle vary widely in morphology depending on their location in the utricle, we sought to determine if afferent morphology might also play a role in spike train irregularity. Although we found that morphology itself is not a factor in determining spike train variability, we did find that spike train variability can be affected by afferent/hair cell connectivity.

Morphological reconstructions of bouton afferents from the lateral extrastriolar (LES), striola, juxtastriola and medial extrastriolar (MES) regions of turtle utricle were done with Neurolucida. Models based on these reconstructions were constructed for 16 afferents. Voltage-dependent conductance distributions in different regions (axon, soma, terminal arbor, varicosities, nodes, myelin) were identical in each cell model. Given the locations of synapses in the terminal arbor and the density of hair cells in each region, we were able to estimate the number of synapses formed between individual hair cells and an afferent. In the LES, striola and juxtastriola most synapses on an afferent were formed with unique hair cells, whereas in the MES single hair cells more often made multiple synapses with an afferent. To determine the effect of this difference in connectivity we modeled Poisson release from hair cells to synapses in the terminal arbor to generate spike trains. The coefficient of variation (cv) as a function of interspike interval (ISI) was calculated to estimate spike train variability with different average release rates.

We found that cv as a function of ISI was nearly identical for LES, striola and juxtastriola afferents, but was the same for MES afferents only if connectivity in the MES was assumed to be one afferent synapse per hair cell. However, cv was much larger when the MES afferents were modeled with multiple synapses between an afferent and a hair cell, as calculated from synaptic location and hair cell density. To see if we could make an LES afferent more irregular, we modified connectivity to have more multiple synapses between individual hair cells and the afferent. As predicted, cv increased.

These results suggest that afferent-hair cell connectivity may be a source of spike train irregularity. Given the large number of multiple contacts between a calyx and its presynaptic hair cell, we expect that connectivity contributes to spike train variability in calyx-bearing (calyx and dimorph) afferents.
